# Pan-cancer integrated bioinformatics analysis reveals cuproptosis related gene FDX1 is a potential prognostic and immunotherapeutic biomarker for lower-grade gliomas

**DOI:** 10.3389/fmolb.2023.963639

**Published:** 2023-02-07

**Authors:** Wei Huang, Yuliang Wu, Jihui Zhu, Ning Luo, Chunyan Wang, Shupeng Liu, Zhongping Cheng

**Affiliations:** ^1^ Department of Obstetrics and Gynecology, Shanghai Tenth People’s Hospital, Tongji University, Shanghai, China; ^2^ School of Medicine, Tongji University, Shanghai, China

**Keywords:** cuproptosis, ferredoxin 1, pan-cancer, lower-grade gliomas, tumor immune microenvironment

## Abstract

FDX1 participates in cuproptosis, a copper-dependent cell death mode, which might influence tumor progressions like ferroptosis and pyroptosis. However, the role of FDX1 in tumors remains to be explored. This study investigated FDX1 expression features, and correlations to prognosis, tumor stages, immune microenvironment, and cuproptosis from a pan-cancer perspective based on integrated bioinformatics. FDX1 mRNA and clinical data were obtained from The Cancer Genome Atlas (TCGA), Genotype-Tissue Expression (GTEx), and Broad Institute Cancer Cell Line Encyclopedia (CCLE) databases. Differential expression of FDX1 in tumor stages was performed on GEPIA2.0. Cox proportional hazard regression and survival curve were used to analyze the prognostic value of FDX1. The relationships between FDX1 expression and immune infiltration, immune cells, immune checkpoints, tumor mutation burden (TMB), microsatellite instability (MSI), mismatch repair (MMR), and DNA methyltransferase (DNMT) were explored. GSEA was utilized to find the biological function of FDX1 in LGG. Results showed that FDX1 was abnormally expressed in multiple tumor types and demonstrated variability in various tumor stages. Survival analysis revealed FDX1 predicted poor prognosis in glioma (GBMLGG), brain lower-grade glioma (LGG), and good prognosis in the pan-kidney cohort (KIPAN), and kidney renal clear cell carcinoma (KIRC). Immune correlation analysis suggested FDX1 showed positive correlations to StromalScore, ImmuneScore, ESTIMATEScore in LGG and negative correlation in KIRC. Additionally, positive correlations were observed between FDX1 and immune cells infiltration, immune checkpoints, tumor stemness, homologous recombination deficiency (HRD), and TMB in LGG in the pan-cancer analysis. Validation with CGGA suggested prognostic value and immune correlation of FDX1 in LGG. Specifically, high expression of FDX1 was accompanied by high expression of immune checkpoints such as CD276 (B7-H3), CD274 (PD-L1), PDCD1LG2 (PD-L2), CTLA4, and HAVCR2. These findings illustrated that FDX1 might be considered a potential poor prognosis biomarker and immunotherapy predictor in LGG.

## 1 Introduction

Immunotherapy, such as immune checkpoints and chimeric antigen receptor T (CAR-T), is a novelty cancer treatment (Galluzzi et al., 2018). Response to immunotherapy often relies on the interaction of tumor cells with immune regulation within the tumor microenvironment (TME) (Bejarano et al., 2021). A variety of pathological and physiological processes including cell death can lead to alterations in the tumor microenvironment and thus affect the efficacy of tumor immunotherapy. Currently, cell death modes including pyroptosis, ferroptosis, and necroptosis are involved in the formation of the tumor suppressive immune microenvironment and are potential immunotherapy targets (Gong et al., 2019; Carneiro and El-Deiry, 2020; Du et al., 2021; Zhang et al., 2022). The induction of pyroptosis, ferroptosis, and necroptosis combined with immune checkpoints showed synergistically enhanced antitumor activity, even in immune checkpoint inhibitors-resistant tumors (Tang et al., 2020). As the metal ion-mediated cell death as ferroptosis, cuproptosis might also be related to the tumor immune microenvironment. The relationship of cuproptosis with TME and whether it could be a potential therapeutic target remain unclear.

Ferredoxin 1 (FDX1) is recently found to be a crucial role in regulating cuproptosis, a copper-dependent cell death similar to ferroptosis (Tsvetkov et al., 2019; Peter et al., 2022). As a member of the [2Fe-2S] cluster-containing ferredoxin family, FDX1 is traditionally thought to participate in the reduction of mitochondrial cytochrome P450 enzymes and in the synthesis of various steroid hormones, bile acid, and vitamin D in mammalian mitochondria (Sheftel et al., 2010). Recent studies have revealed that FDX promotes protein lipoylation and facilitates copper binding, which in turn promotes the aggregation of lipoylated proteins and the destabilization of Fe-S cluster proteins, leading to proteotoxic stress and ultimately cell death (Peter et al., 2022). FDX1 acted as a biomarker for elesclomol due to the FDX1 activity and promoted mitochondria-dependent energy metabolism inducing the toxic effect of elesclomol in tumor cells (Tsvetkov et al., 2019). In lung adenocarcinoma, FDX1 was a part of the electron transport chain risk signature predicting prognosis and was a regulator of glucose metabolism, fatty acid oxidation, and amino acid metabolism (Zhang et al., 2021). However, none of its role in tumor as well as immunotherapy is reported. More functions of FDX1 and cuproptosis in tumors remain to be explored.

In the current study, we explored the role of FDX1 in human pan-cancer by transcritomic analysis. The expression pattern of FDX1 and its prognostic value, and its correlation with the immune microenvironment were explored based on TCGA and GTEx datasets. FDX1 was differently expressed in most cancer and its expression was associated with immune characteristics and other tumor characteristics including Tumor Mutation Burden (TMB), Microsatellite Instability (MSI), Mismatch Repair (MMR), and DNA methyltransferase (DNMT) in pan-cancer. Specially, we found FDX1 was a poor prognosis predictor and correlated to the suppressive immune microenvironment in lower-grade gliomas with the validation of CGGA. Our study revealed that FDX1 was a potential prognostic and immunotherapeutic biomarker.

## 2 Materials and methods

### 2.1 Data collection

The mRNA expression data in various tumor types were obtained from The Cancer Genome Atlas (TCGA) database (https://portal.gdc.cancer.gov/). The mRNA expression profile with normal tissue ©was extracted from Genotype-Tissue Expression (GTEx) database (https://gtexportal.org/home/datasets) to supply normal tissue RNA-seq transcriptome data lacking in TCGA. FDX1 expression profile of tumor cell lines was obtained from Broad Institute Cancer Cell Line Encyclopedia (CCLE) database (https://portals.broadinstitute.org/ccle/data). All clinical information in pan-cancer came from TCGA Pan-Cancer of UCSC dataset (https://xenabrowser.net/). To verify the result in TCGA-LGG, the mRNA expression and clinical data were obtained from the Chinese Glioma Genome Atlas (CGGA) database (http://www.cgga.org.cn/). We utilized the “mRNAseq_325” dataset and defined the WHOII and WHO III as LGG to research the role of FDX1 in LGG (Liu et al., 2018). The mRNA data was removed of the expression value of zero, duplicated, and filtered the data with a follow-up time was less than 30 days. Tumor samples with less than three must be deleted in all pan-cancer analyses.

### 2.2 The prognostic value of FDX1 and its clinical characteristics

The differential expression of FDX1 in various tumor stages was analyzed by GEPIA2.0 (http://gepia2.cancer-pku.cn/#index) (Tang et al., 2019). We used the “coxph” function of R package “survival” to establish Cox proportional hazards regression model was evaluated the correlation between FDX1 expression and overall survival (OS), disease-specific survival (DSS), and progression-free interval (PFI) according to TCGA Pan-cancer dataset. The cox regression results were shown by the forest plot. The survival analysis was utilized by R package “survminer” and “survival” and the “high” and “low” subgroup was depended on the cutoff value of FDX1 expression, and was shown by the Kaplan-Meier plot. The prognostic value of FDX1 and its clinical characteristics in CGGA was obtained in the CGGA online website using the analysis function.

### 2.3 Correlation between FDX1 expression and immune characteristics

We calculated the ESTIMATEScore, ImmuneScore, and StromalScore by the R package “ESTIMATE” (Yoshihara et al., 2013) and the immune cells infiltration including B cell, CD4^+^T cell, CD8^+^T cell, neutrophil, macrophage, and dendritic cell (DC) based on “TIMER” algorithm of R package “IOBR” according to TCGA-LGG, TCGA-GBMLGG, TCGA-KIRC, TCGA-KIPAN, CGGA-LGG, CGGA-GBM datasets (Li et al., 2017; Zeng et al., 2021). The correlation between FDX1 expression and ESTIMATEScore, ImmuneScore, StromalScore, and immune cells was assessed by Pearson coefficient.

### 2.4 Gene set enrichment analysis

The mRNA expression data obtained from TGCA-LGG and CGGA-LGG databases was divided into high and low subgroups based on the median value of FDX1 expression and then analyzed by R package “limma” that calculated the fold change value (Ritchie et al., 2015). The biological function of FDX1 involved in biological process (BP) pathways, KEGG pathways, and Hallmark pathways were performed by GSEA software (http://software.broadinstitute.org/gsea/index.jsp) according to the log2Fold change value.

### 2.5 Statistical analysis

Differential expression of FDX1 in various tissue and cancer cell lines was used by the Kruskal–Wallis test, and between tumor and normal tissue were evaluated by *t*-test. Meanwhile, the ANOVA test and *t*-test assessed the expression of FDX1 in different grades of glioma. In the survival and cox regression analysis procedure, Log-rank *p*-value, 95% confidence interval, and hazard ratios (HRs) were calculated. All correlation analysis visualized by R package “psych.” *p* < 0.05 was considered significant in all analysis results.

## 3 Results

### 3.1 The expression of FDX1 pattern and its clinical characteristics in pan-cancer

**FIGURE 1:**
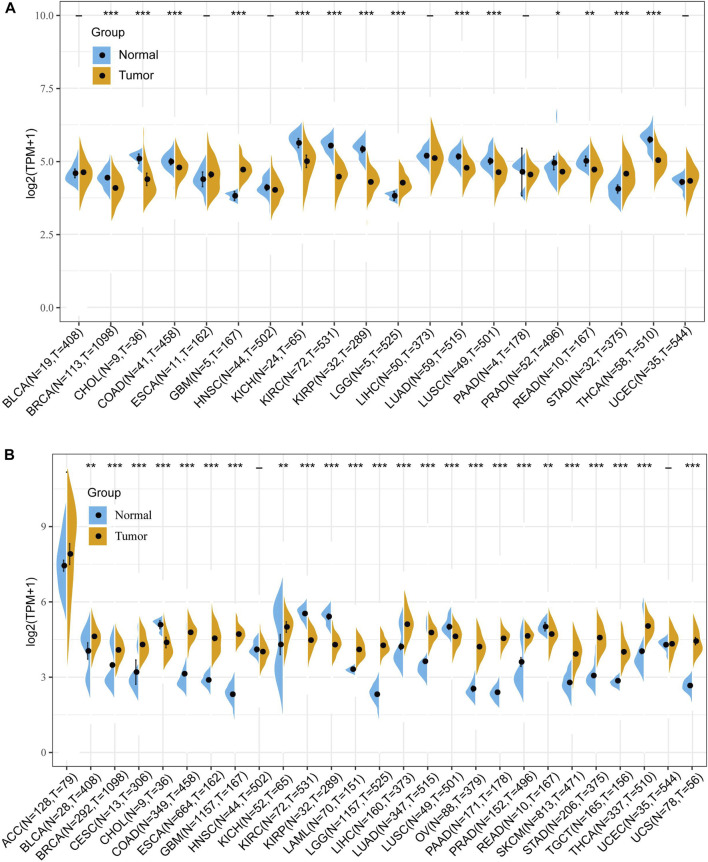
Differential expression of FDX1 in human pan-cancer according to The Cancer Genome Atlas (TCGA) and Genotype-Tissue Expression (GTEx) databases. **(A)** The mRNA expression of FDX1 in pan-cancer based on TCGA database. **(B)** The mRNA expression of FDX1 in pan-cancer based on TCGA and GTEx databases. (*t*-test, *p* < 0.05 was considered significant, *p < 0.05, **p < 0.01, ***p < 0.001, N, Normal tissue; T, Tumor tissue).

First, to investigate the differential expression of FDX1 in tumor and normal tissue, the FDX1 mRNA expression was obtained from TCGA database. The differential expression analysis result demonstrated that the FDX1 was highly expressed in tumor tissues than in normal tissues in glioblastoma multiforme (GBM), brain lower-grade glioma (LGG), stomach adenocarcinoma (STAD) (Figure 1A). Reduced FDX1 expression in tumor tissues was observed in breast invasive carcinoma (BRCA), cholangiocarcinoma (CHOL), colon adenocarcinoma (COAD), kidney chromophobe (KICH), renal clear cell carcinoma (KIRC), kidney renal papillary cell carcinoma (KIRP), lung adenocarcinoma (LUAD), lung squamous cell carcinoma (LUSC), prostate adenocarcinoma (PRAD), rectum adenocarcinoma (READ), thyroid carcinoma (THCA) (Figure 1A). Then, we analyzed the expression of FDX1 in normal tissue according to the GTEx dataset. The result suggested that FDX1 was highly expressed in the adrenal gland, liver, and thyroid and lowly expressed in blood, brain, and pancreas (Supplementary Figure S1A). Then, the result of FDX1 expression in tumor cell lines showed that it was highest expressed in the intestine, stomach, and hematopoietic and lymphoid and lowest expressed in pleura and upper aerodigestive tract (Supplementary Figure S1B) based on the CCLE database.

Due to the small number of normal samples in the TCGA database, we further integrated TCGA and GTEx databases to assess the differential expression of FDX1. As shown in Figure 1B, FDX1 was upregulated in Bladder Urothelial Carcinoma (BLCA), BRCA, cervical squamous cell carcinoma and endocervical adenocarcinoma (CESC), COAD, esophageal carcinoma (ESCA), GBM, KICH, acute myeloid leukemia (LAML), LGG, liver hepatocellular carcinoma (LIHC), LUAD, ovarian serous cystadenocarcinoma (OV), pancreatic adenocarcinoma (PAAD), PRAD, skin cutaneous melanoma (SKCM), stomach adenocarcinoma (STAD), testicular germ cell tumors (TGCT), THCA, and uterine carcinosarcoma (UCS), and was downregulated in CHOL, KIRC, KIRP, LUSC, and READ. No difference in expression of FDX1 in adrenocortical carcinoma (ACC), HNSC, and uterine corpus endometrial carcinoma (UCEC) (Figure 1B).

**FIGURE 2:**
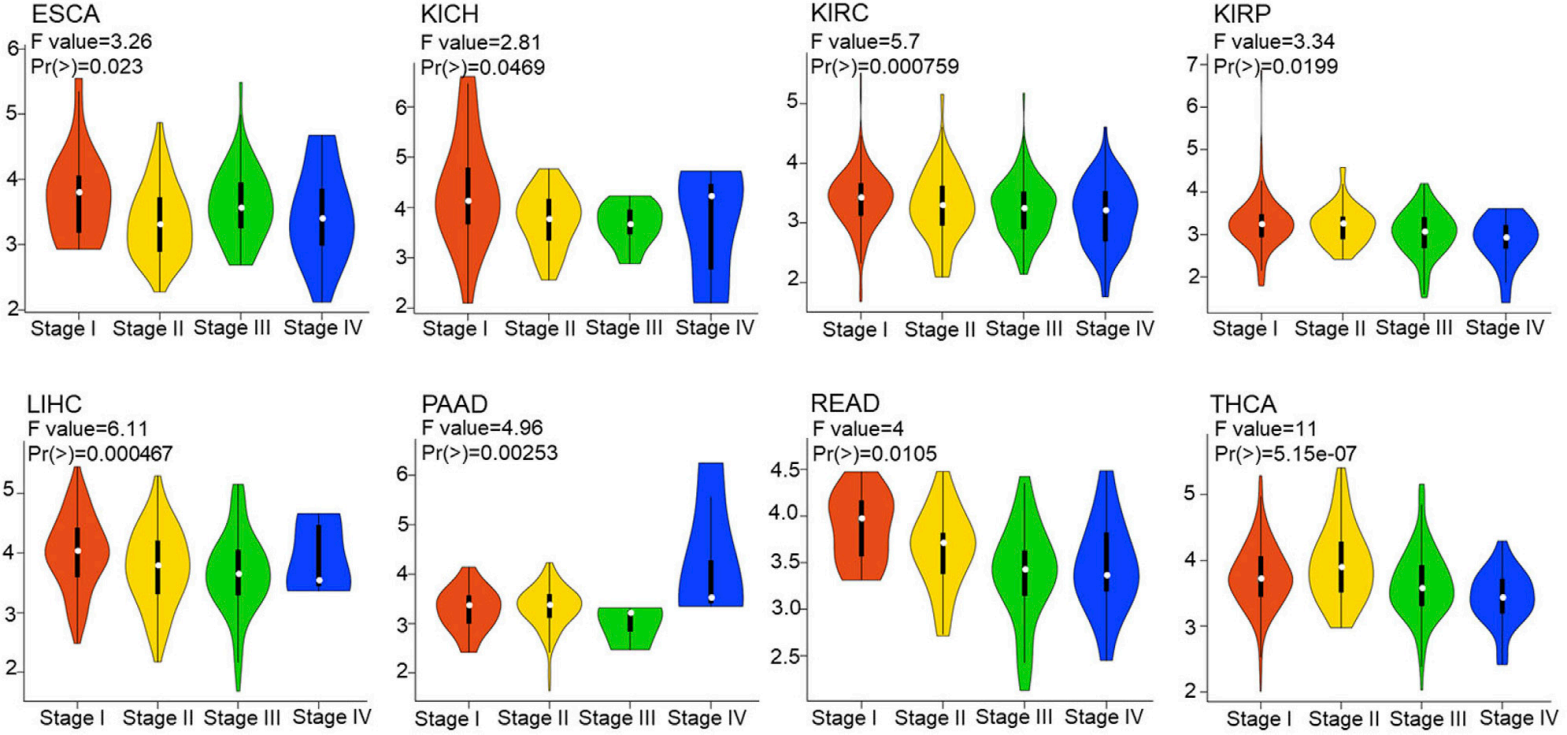
Correlation between FDX1 expression and tumor stages. The correlation between FDX1 expression and tumor stages in pan-cancer based on Gene Expression Profile Interactive Analysis 2.0 (GEPIA 2.0). [one way ANOVA test, Pr(>F) < 0.05 was considered significant. The larger the F value and the smaller the Pr (>F) value, the greater difference in characteristics between groups.]

Furthermore, we explored FDX1 expression in different stage in pan-cancer according to GEPIA2.0. The result illustrated FDX1 was differentially expressed in THCA [F value = 11, Pr (>F) = 5.15e-07], LIHC [F value = 6.11, Pr (>F) = 0.000467], KIRC [F value = 5.7, Pr (>F) = 0.000759], PAAD (F value = 4.96, *p* = 0.00253), READ [F value = 4, Pr (>F) = 0.0105], KIRP [F value = 3.34, Pr (>F) = 0.0199], ESCA [F value = 3.26, Pr (>F) = 0.023], and KICH [F value = 2.81, Pr (>F) = 0.0469] (Figure 2). No differential expression of FDX1 in other tumor’s stage including ACC, BLCA, BRCA, CESC, etc. (Supplementary Figure S2).

**FIGURE 3:**
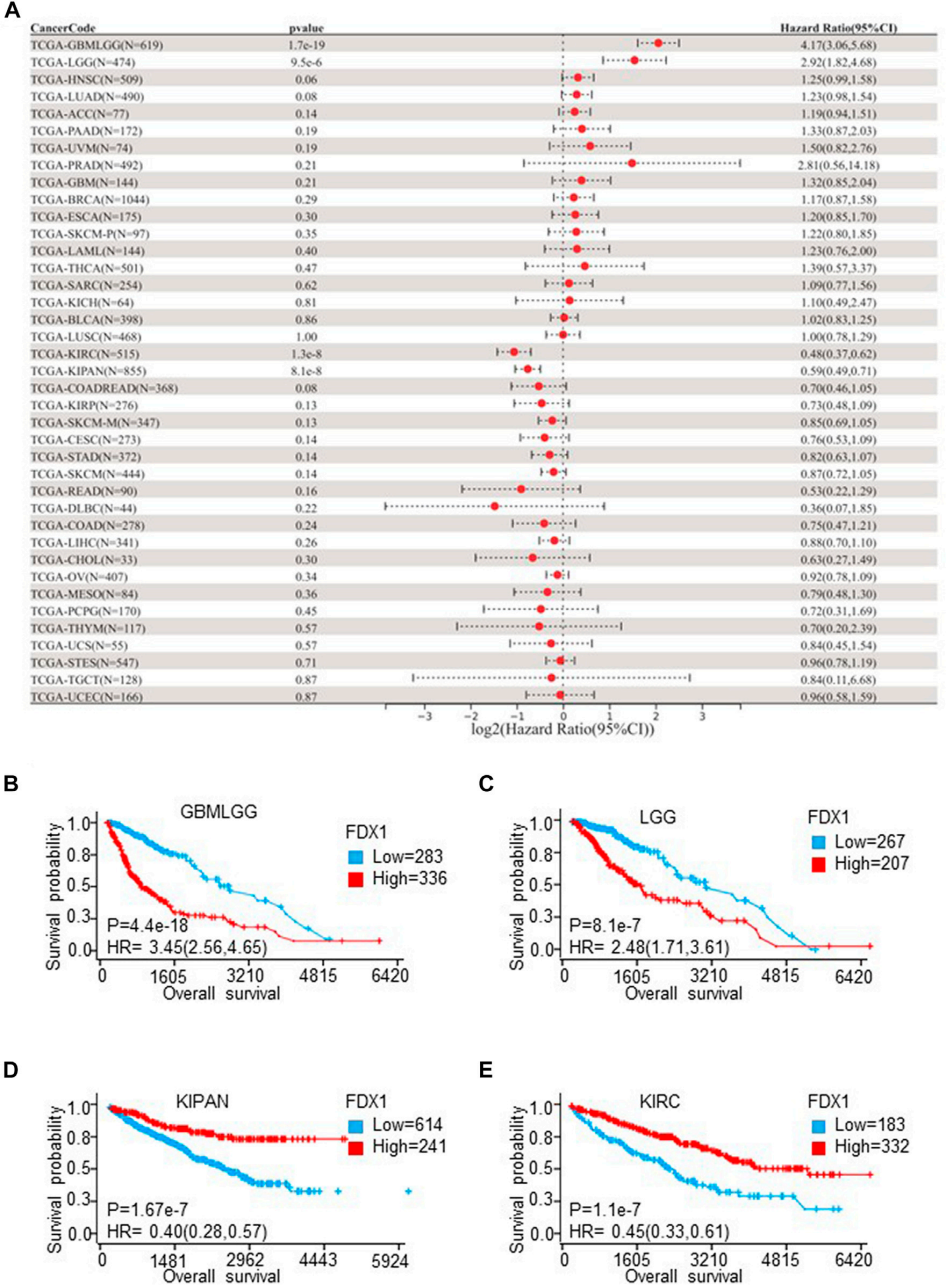
The overall survival (OS) analysis of FDX1 in different cancer types according to TCGA Pan-Cancer database. **(A)** The relationship between FDX1 expression and hazard ratio (HR) was shown by the forest plot. **(B–E)** Kaplan-Meier plot shows the relationship between FDX1 expression and OS in LGGGBM, LGG, KIPAN, and KIRC. (LGGGBM: glioma, GBM: brain lower-grade glioma, KIPAN: pan-kidney cohort, KIRC: renal clear cell carcinoma. *p* < 0.05 was considered significant.)

Univariate Cox proportional hazards models were employed to explore the survival predictive value of FDX1. The prognostic value of FDX1 was estimated by OS, DSS, and PFI. OS results showed that expression of FDX1 was risk factor in glioma (GBMLGG) (HR = 4.17, *p* = 1.7e-19), LGG (HR = 2.92, *p* = 9.5e-6), and was protective factor in KIRC (HR = 0.48, *p* = 1.3e-8) and pan-kidney cohort (KIPAN) (HR = 0.59, *p* = 8.1e-8) (Figure 3A). DSS results demonstrated FDX1 was correlated to DSS in GBMLGG (HR = 4.64, *p* = 2.0e-20), LGG (HR = 2.97, *p* = 1.5e-5), SKCM (HR = 1.72, *p* = 0.04), KIRC (HR = 0.38, *p* = 6.1e-10), KIPAN (HR = 0.50, *p* = 7.6e-9), KIRP (HR = 0.56, *p* = 0.03), and THYM (HR = 0.33, *p* = 0.03) (Supplementary Figure S3A). The analysis results of PFI also illustrated that FDX1 was also a risk factor in ACC (HR = 1.34, *p* = 4.2e-3), and a protective factor in THCA (HR = 0.46, *p* = 1.6e-3), MESO (HR = 0.54, *p* = 0.03) (Supplementary Figure S3B). Then, the Kaplan-Meier plot showed that high expression of FDX1 was associated with poor prognosis in GMBLGG (HR = 3.45, *p* = 4.4e-18) and LGG (HR = 2.48, *p* = 8.1e-7), and good prognosis in KIPAN (HR = 0.4, *p* = 1.67e-7) and KIRC (HR = 0.45, *p* = 1.1e-7) (Figures 3B–E). Meanwhile, we analyzed the relationship between the expression of FDX1 and the prognosis of GBM, KICH, and KIRP. The result displayed that high expression of FDX1 was related to poor prognosis in GBM (HR = 1.4, *p* = 0.11), and KICH (HR = 3.53, *p* = 0.05), and good prognosis in KIRP (HR = 0.54, *p* = 0.04) (Supplementary Figure S4A). In conclusion, these results notably reflected that high expression of FDX1 was an important risk factor in LGG and a protective factor in KIRC, which affects the prognosis of GBMLGG and KIPAN.

### 3.2 Correlation between FDX1 expression and immune characteristics in pan-caner

**FIGURE 4:**
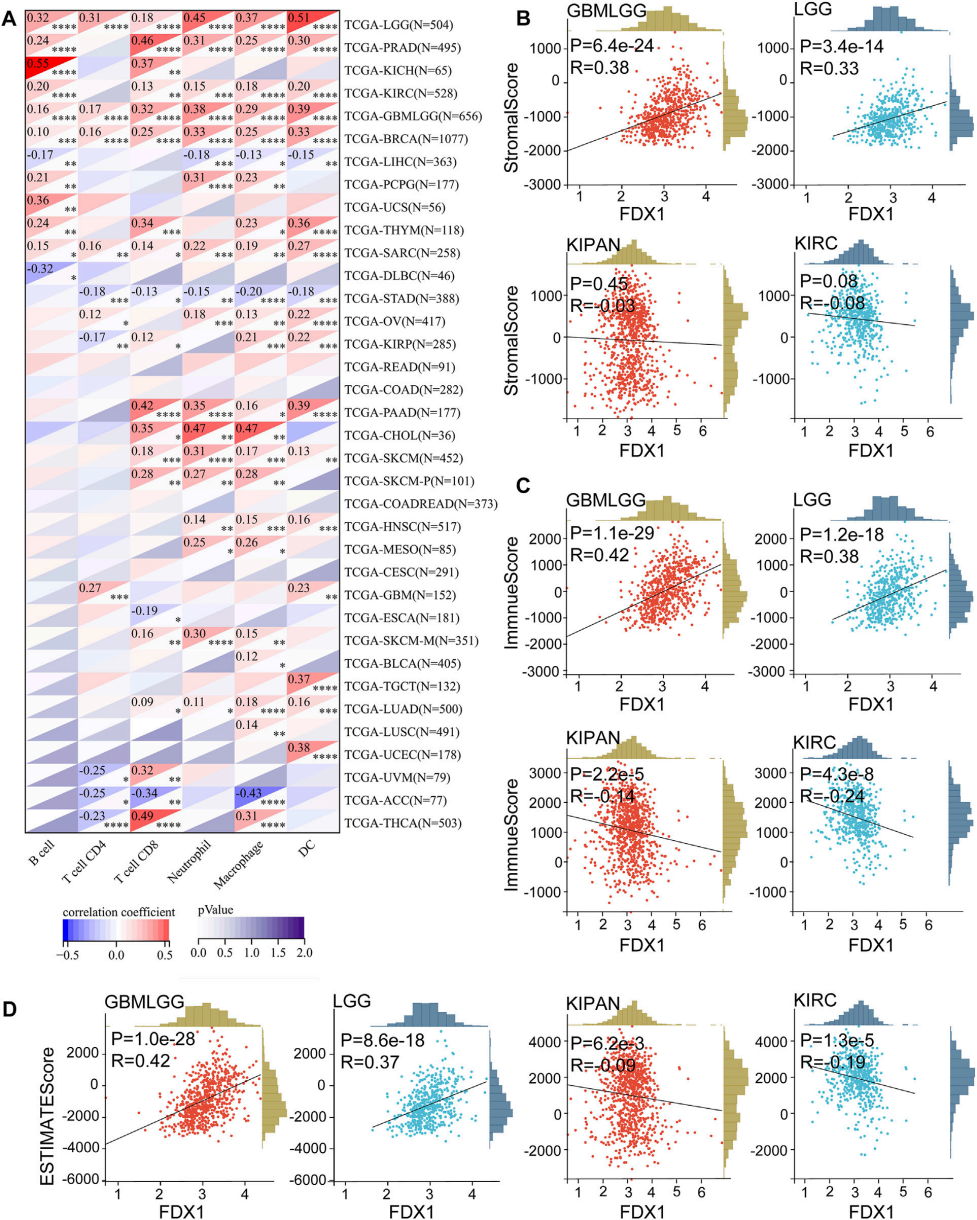
Correlation between FDX1 and immune cells infiltration in pan-cancer, StromalScore, ImmuneScore, ESTIMATEScore in LGGGBM, LGG, KIPAN, and KIRC. **(A)** The FDX1 expression was associated with B cell, CD4^+^T cell, CD8^+^T cell, neutrophil, macrophage, and dendritic cell (DC) based on the TIMER algorithm. **(B)** The FDX1 expression was associated with StromalScore in LGGGBM, LGG, KIPAN, and KIRC. **(C)** The FDX1 expression was associated with ImmuneScore in LGGGBM, LGG, KIPAN, and KIRC. **(D)** The FDX1 expression was associated with ESTIMATEScore in LGGGBM, LGG, KIPAN, and KIRC. (*p* < 0.05 was considered significant, all correlation analysis was shown by Pearson coefficient.)

To explore whether cuproptosis is related to the tumor immune microenvironment, we explored the correlation of cuproptosis core protein FDX1 with immunity. First, we explored the correlation between FDX1 and immune cell infiltration with the TIMER algorithm (Li et al., 2017). FDX1 expression was positively correlated to B cell, CD4^+^T cell, CD8^+^T cell, neutrophil, macrophage, and dendritic cell (DC) infiltration in multiple cancers, especially in LGG (Figure 4A). Besides CD4^+^T cell, FDX1 was positively related to other immune cells in PRAD, KIRC (Figure 4A). Otherwise, FDX1 expression was negatively correlated to CD4^+^T cell, CD8^+^T cell, neutrophil, macrophage, and dendritic cell in STAD (Figure 4A). The correlation between FDX1 expression and tumor environment based on the ESTIMATE analysis was further investigated (Yoshihara et al., 2013). Strongly positive correlations between FDX1 expression and StromalScore, ImmuneScore, and ESTIMATEScore were observed in LGG and GBMLGG (Figures 4B–D), while negative correlations were observed in KIRC, KIPAN (Figures 4B–D). However, there was no significant correlation in GBM (Supplementary Figure S4B).

**FIGURE 5:**
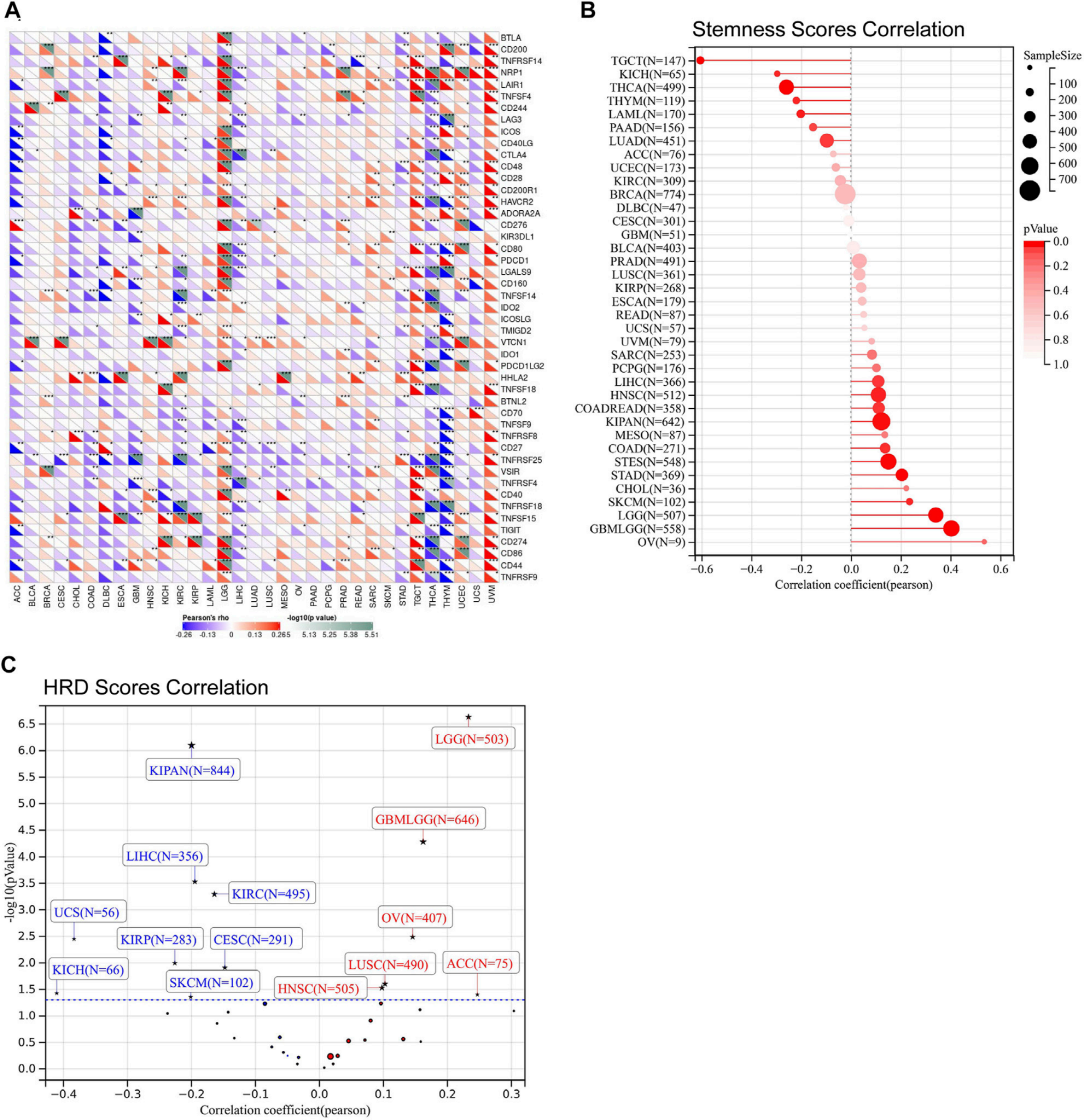
The relationship between FDX1 expression and immune checkpoints, stemness scores obtained from DNA methylation data, and homologous recombination deficiency (HRD). **(A)** Correlation between FDX1 expression and immune checkpoints. **(B)** Correlation between FDX1 expression and stemness scores. **(C)** Correlation between FDX1 expression and HRD [Positive correlation tumor type including GBMLGG (N = 646, R = 0.16, *p* = 0.00005), LGG (N = 503, R = 0.23, *p* = 2.30e-7), HNSC (N = 505) (R = 0.10, *p* = 0.03), LUSC (N = 490, R = 0.10, *p* = 0.02), OV (N = 406, R = 0.13, *p* = 0.008), ACC (N = 75, R = 0.25, *p* = 0.03) and Negative correlation tumor type including CESC (N = 291, R = −0.15, *p* = 0.01), KIRP(N = 283, R = −0.23, *p* = 0.01), KIPAN (N = 844, R = −0.20, *p* = 7.85e-7), KIRC (N = 495, R = −0.16, *p* = 0.0005), LIHC (N = 356, R = −0.19, *p* = 0.0002), SKCM (N = 102, R = −0.20, *p* = 0.04), UCS (N = 56, R = −0.38, *p* = 0.005), KICH (N = 66, R = −0.41, *p* = 0.04)]. (All correlation analysis was shown by Pearson coefficient, *p* < 0.05 was considered significant, *p < 0.05, **p < 0.01, ***p < 0.001.)

Immune checkpoints, tumor stemness, homologous recombination deficiency (HRD), TMB, MSI, DNMT, and MMR were important evaluation signatures for immunotherapy and adjuvant therapy, which could predict treatment effectiveness and the prognosis of patients (Chiappinelli et al., 2016; Luchini et al., 2019; Clara et al., 2020; Morand et al., 2021). Thus, we determined the correlations between FDX1 expression and the signatures. FDX1 expression was positively correlated to most immune checkpoints in LGG, TGCT, and UVM, and was negatively correlated to most immune checkpoints in THCA, and THYM (Figure 5A). Specifically, CD276 (B7-H3), CD274 (PD-L1), PDCD1LG2 (PD-L2), CTLA4, HAVCR2, and PDCD1 (PD-1) were strongly correlated to FDX1 in LGG (Figure 5A). In addition, tumor stemness correlation analysis suggested that FDX1 expression was correlated to stemness score across multiple tumor types (Figure 5B). Notably, a strongly positive correlation was observed in GBMLGG and LGG, while no significant correlation was observed in GBM (Figure 5B). HRD correlation analysis revealed that FDX1 expression was significantly related to HRD in 14 tumor types (Figure 5C). Similarly, a strongly positive correlation was observed in GBMLGG (*p* = 0.00005, R = 0.16) and LGG (P= *p*=2.30e-7, R = 0.23), while no significant correlation was observed in GBM (Figure 5C). Meanwhile, a strongly negative correlation was observed in KIPAN (*p* = 7.85e-7, R = −0.19) and KIRC (*p* = 0.0005, R = −0.16) (Figure 5C).

**FIGURE 6:**
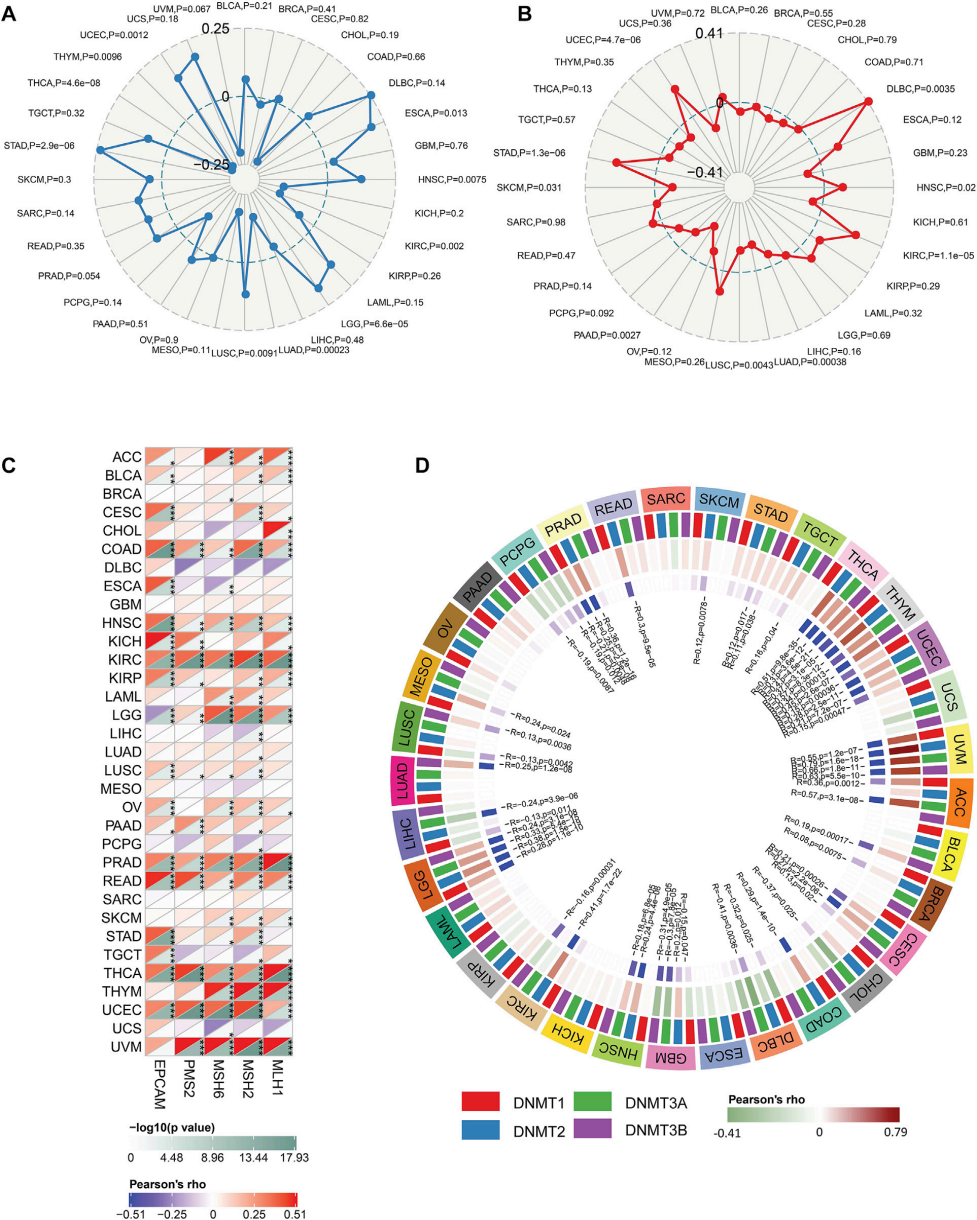
The correlation between FDX1 and tumor Mutation Burden (TMB), microsatellite instability (MSI), mismatch repair (MMR), and DNA methyltransferase (DNMT). **(A)** The correlation between FDX1 expression and TMB. **(B)** The correlation between FDX1 expression and MSI. **(C)** The correlation between FDX1 expression and MMR genes (MLH1, MSH2, MSH6, and EpCAM). **(D)** The correlation between FDX1 expression and DNMT (DNMT1, DNMT2, DNMT3A, and DNMT3B). (TMB and MSI were analyzed by Spearman coefficient, other correlation analysis was shown by Pearson coefficient, *p* < 0.05 was considered significant, *p < 0.05, **p < 0.01, ***p < 0.001.)

Besides, our results exhibited that FDX1 was closely related to TMB, MSI, DNMT, and MMR across multiple tumor types. In the analysis procedure of TMB, results showed FDX1 was positively correlated to TMB in ESCA, HNSC, LGG, LUSC, STAD, and UCEC and negatively related to TMB in KIRC, LUAD, THYM, THCA (Figure 6A). A positive correlation between FDX1 expression and MSI was observed in DLBC, HNSC, KIRC, STAD, and UCEC (Figure 6B). And, FDX1 with MSI was a significant negative correlation in LUAD, LUSC, PAAD, and SKCM (Figure 6B). We researched the association of FDX1 expression and MMR based on five genes including MLH1, MSH2, MSH6, PMS2, and EpCAM. FDX1 expression showed a close correlation to MMR in ACC, BLCA, CESC, COAD, HNSC, KICH, KIRC, KIRP, LGG, LUSC, OV, PRAD, READ, THCA, UCEC, and UVM (Figure 6C). The correlation between FDX1 and DNA methylation was also studied in that the latter was vital for epigenetics. We analyzed the relationship between FDX1 and four methylation transferases containing DNMT1, DNMT2, DNMT3A, and DNMT3B. The results suggested that FDX1 was positively related to DNMT1, DNMT2, DNMT3A, and DNMT3B in THYM, UCEC, UVM, and LGG (Figure 6D). Otherwise, FDX1 has negatively associated with other methylation transferases in GBM apart from DNMT2 (Figure 6D).

### 3.3 Validation of FDX1 expression and associated tumor characteristics in brain lower-grade glioma

**FIGURE 7:**
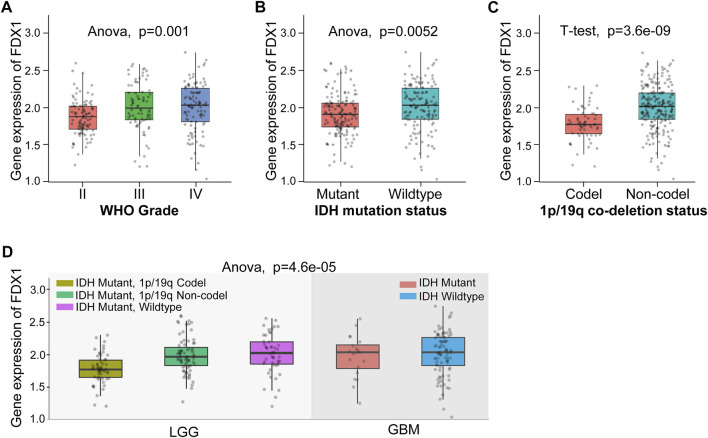
The differential expression of FDX1 in brain lower-grade glioma (LGG) according to Chinese Glioma Genome Atlas (CGGA) database. **(A)** Differential expression of FDX1 in different grades in LGG. **(B)** Differential expression of FDX1 in LGG depended on isocitrate dehydrogenase (IDH) mutation status. **(C)** Differential expression of FDX1 in LGG depended on 1p/19q co-deletion status. **(D)** Differential expression of FDX1 in LGG depended on IDH mutation status combined with 1p/19q co-deletion status. (All statistical methods were shown in the figure, *p* < 0.05 was considered significant.)

Based on the above results, we found the FDX1 expression demonstrated prominent relevance to prognosis, immune infiltration, and immunotherapy-related factors including immune checkpoints, tumor stemness, HRD, TMB, MMR, and DNMT in LGG. Therefore, we further verify the tumor-promoting role of FDX1 in LGG according to Chinese Glioma Genome Atlas (CGGA) database. Aiming at clinical characteristics, we found there was a significant differential FDX1 expression in WHO Grade II, WHO Grade III, and WHO Grade IV (*p* = 0.001), and FDX1 was highly expressed in WHO IV (GBM) compared with WHO II, III (LGG) (Figure 7A), consistent with the result in TCGA-GBM and TCGA-LGG (Figures 1A, B). Isocitrate dehydrogenase (IDH) mutation status and 1p/19q co-deletion status are important factors to estimate the diagnosis, prognosis, and treatment condition of glioma. We found that FDX1 was highly expressed in IDH wildtype patients (*p* = 0.0052) and 1p/19 non-codeletion patients (*p* = 3.6e-09) (Figures 7B, C). Subsequently, we also illustrated that FDX1 was expressed differentially in different glioma molecular subtypes including LGG IDH mutant-1p/19q codel, LGG IDH mutant-1p/19q non-codel, LGG IDH mutant-wildtype and GBM IDH mutant, IDH-wildtype (*p* = 4.6e-05) (Figure 7D), suggesting a differential FDX1 expression in glioma molecular subtypes and indicating a potential function of FDX1 in prognosis and treatment of glioma.

**FIGURE 8:**
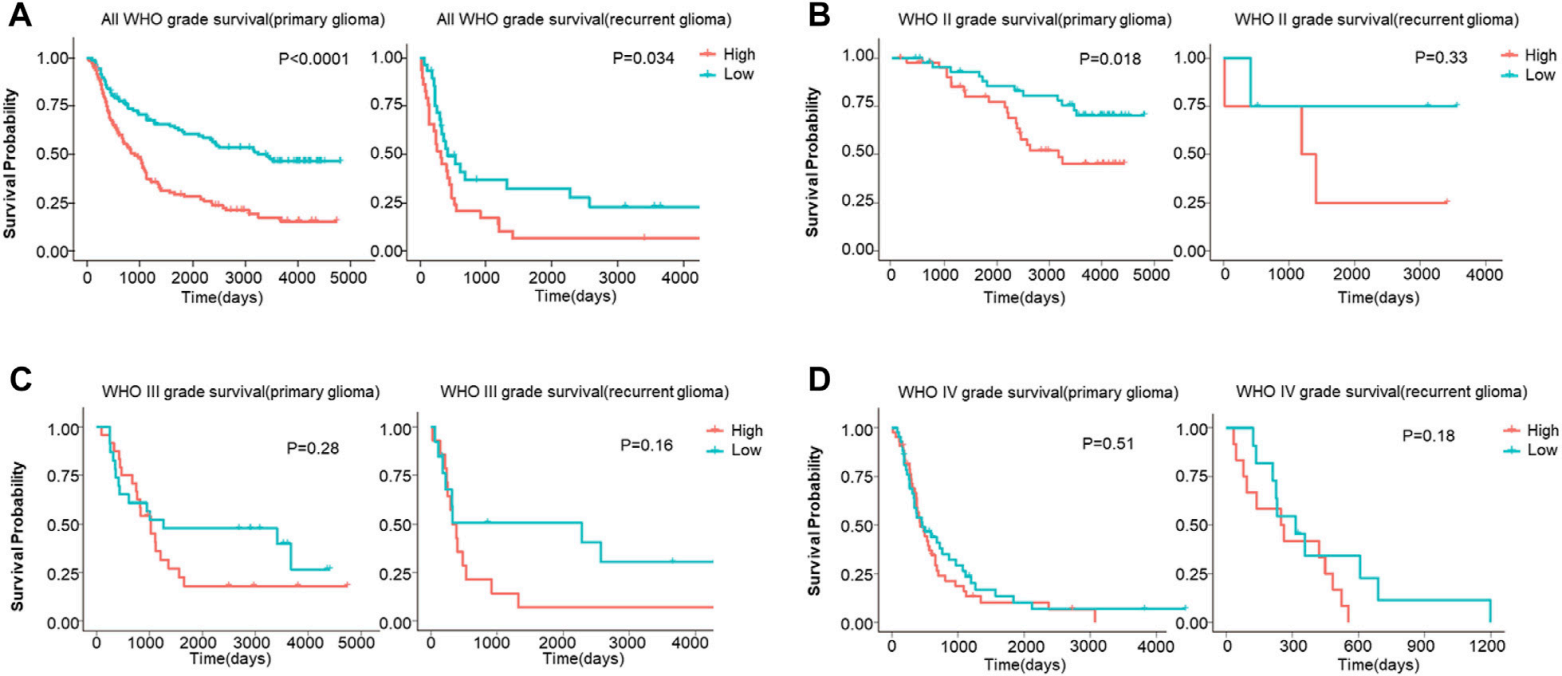
The overall survival (OS) analysis of FDX1 expression in glioma based on CGGA database. **(A)** FDX1 expression was related to OS in all WHO grade of primary glioma and recurrent glioma. **(B–C)** FDX1 expression was related to OS in LGG of primary glioma and recurrent glioma. **(D)** FDX1 expression was related to OS in GBM of primary glioma and recurrent glioma. (LGG: WHOII and WHO III grades, GBM: WHO IV grade, *p* < 0.05 was considered significant.)

Then, the survival analysis displayed a high expression of FDX1 was a poor prognostic factor for all WHO grade survival in primary glioma (*p* < 0.0001) or recurrent glioma (*p* = 0.034) (Figure 8A). In different grades, our results illustrated high expression of FDX1 as a risk factor for WHO II primary glioma (LGG) (*p* = 0.018) (Figure 8B), and the result was similar to TCGA-LGG (Figure 3C). However, this result was not turned out in WHO III (LGG) (*p* = 0.28), WHO IV primary glioma (GBM) (*p* = 0.51), and WHO II (LGG) (*p* = 0.33), WHO III (LGG) (*p* = 0.16) or WHO IV (GBM) recurrent glioma (*p* = 0.18) (Figures 8B–D). The results verified the survival analysis result in TCGA.

**FIGURE 9:**
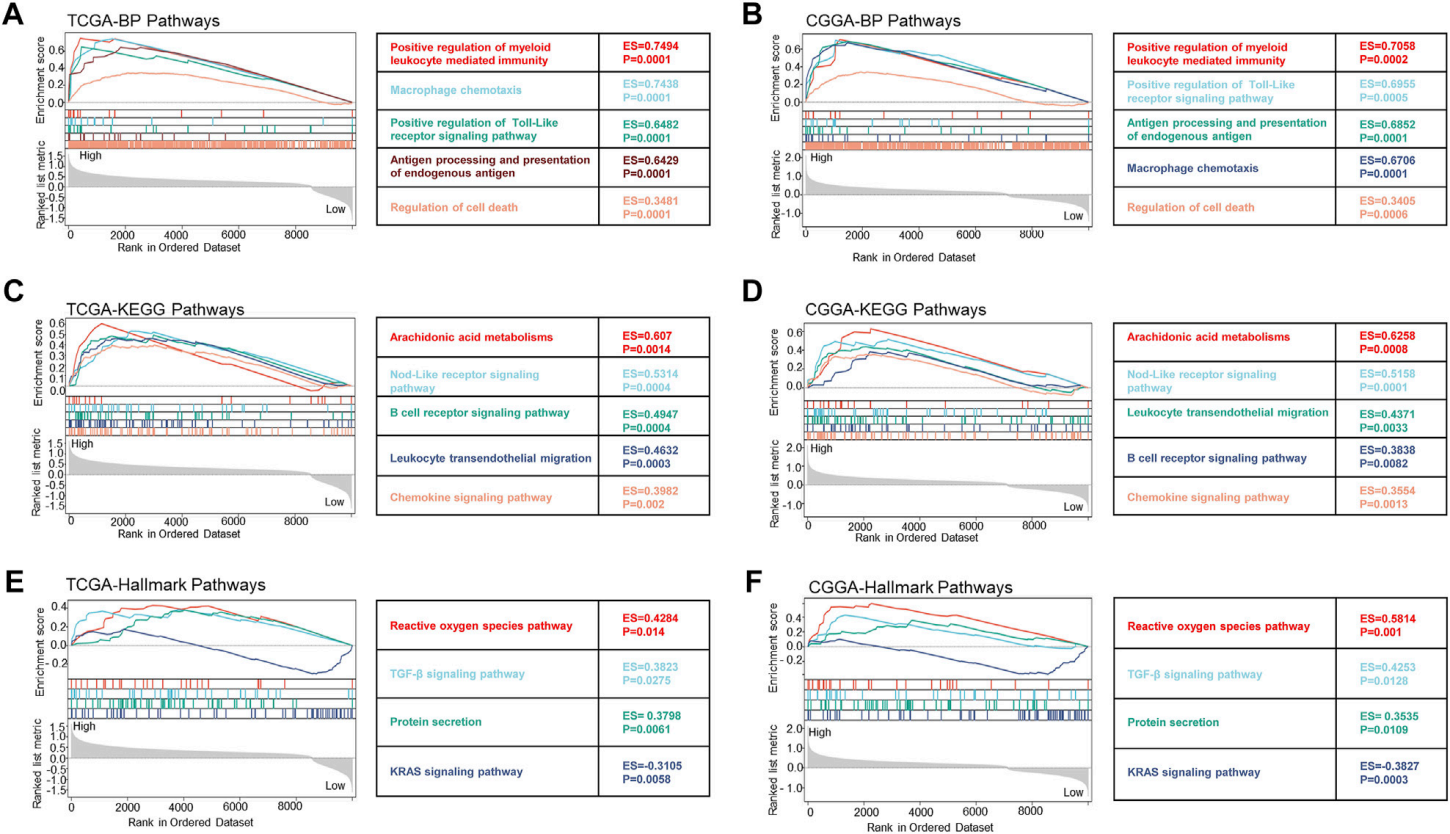
The biological function of FDX1 gene was shown in biological process (BP) pathways, KEGG pathways, and Hallmark pathways in LGG according to TCGA and CGGA datasets. **(A,B)** Relationship between FDX1 expression and BP pathways. **(C,D)** Relationship between FDX1 expression and KEGG pathways. **(E,F)** Relationship between FDX1 expression and Hallmark pathways. (All analytical methods have relied on GSEA algorithm, ES, enrichment score, *p* < 0.05 was considered significant.)

To clarify the specific mechanism of FDX1 affected LGG survival, GSEA analysis suggested the key biological function of FDX1 according to the high and low subgroups in TCGA-LGG and CGGA-LGG (WHO grade II, III). The biological process results demonstrated that the high FDX1 expression subgroup was mainly involved in immune-mediated pathways including positive regulation of myeloid leukocyte meditated immunity, macrophage chemotaxis, antigen processing and presentation of endogenous antigen, and inflammation response, such as positive regulation of Toll-Like receptor signaling pathway and regulation of cell death based on TCGA-LGG and CGGA- LGG datasets (Figures 9A, B). Meanwhile, KEGG pathways showed a high FDX1 expression subgroup participated in immune-mediated pathways containing leukocyte transendothelial migration and B cell receptor, inflammation-related pathways including Nod-Like receptor signaling pathway, and chemokine signaling pathway, and metabolism-related pathways such as arachidonic acid metabolism (Figures 9C, D). Subsequently, analysis of Hallmark pathways is beneficial to research tumor-related pathways. Our results suggested that high expression of the FDX1 group mainly referred to reactive oxygen species (ROS) pathway, TGF-β signaling pathway, and protein secretion. The low FDX expression was involved in the KRAS signaling pathway according to TCGA-LGG and CGGA-LGG databases (Figures 9E, F). Notably, these results reflected the biological function of FDX1 was involved in immune-mediated pathways, inflammation, metabolism, and cell death-related pathways.

**FIGURE 10:**
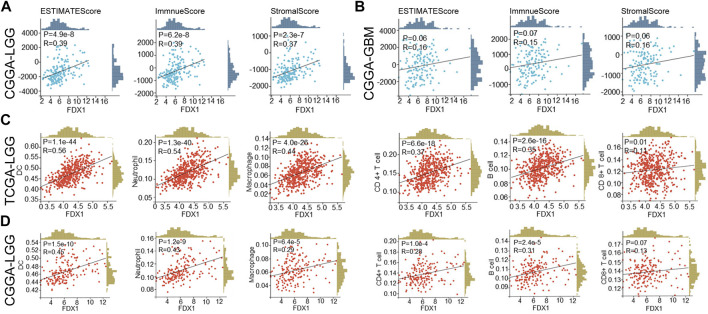
FDX1 was correlated to immune cells infiltration in LGG based on TCGA and CGGA databases and StromalScore, ImmuneScore, and ESTIMATEScore in CGGA database. **(A)** The correlation between FDX1 expression and StromalScore, ImmuneScore, and ESTIMATEScore in CGGA-LGG database. **(B)** The correlation between FDX1 expression and StromalScore, ImmuneScore, and ESTIMATEScore in CGGA-GBM database. **(C)** The correlation between FDX1 expression and immune cells infiltration in TCGA-LGG database. **(D)** The correlation between FDX1 expression and immune cells infiltration in CGGA-LGG database. [Immune cells: B cell, CD4^+^T cell, CD8^+^T cell, neutrophil, macrophage, and dendritic cell (DC), all correlation analyses were shown by Pearson coefficient, *p* < 0.05 was considered significant.]

We further verified the correlation between FDX1 expression and immune infiltration in the CGGA dataset. Results suggested FDX1 was positively correlated to ESTIMATEScore (R = 0.39, *p* = 4.9e-8), ImmuneScore (R = 0.39, *p* = 6.2e-8), and StromalScore (R = 0.37, *p* = 2.3e-7) in CGGA-LGG database (Figure 10A), consistent with the result in TCGA-LGG (Figures 4B–D). No difference between FDX1 and ESTIMATEScore (R = 0.16, *p* = 0.06), ImmuneScore (R = 0.15, *p* = 0.07), and StromalScore (R = 0.16, *p* = 0.06) in CGGA-GBM database (Figure 10B), also consistent with the result in TCGA-GBM (Supplementary Figure S4B). Then, the correlation analysis demonstrated FDX1 expression positively correlated to DC, neutrophil, macrophage, CD4^+^T cell, and B cell in both TCGA-LGG (Figure 10C) and CGGA-LGG datasets (Figure 10D). Otherwise, FDX1 expression demonstrated positive correlations to many immune checkpoints in the CGGA-LGG dataset, among which CD274, CD276, HAVCR2, ICOS, and PDCD1LG2 were positively related to FDX1 expression in both TCGA-LGG and CGGA-LGG datasets (Table 1).

**TABLE 1:** Correlations between the FDX1 expression and immune checkpoints in TCGA-LGG and CGGA-LGG datasets.

Immune checkpoint	TCGA		CGGA	
	Cor	*p*-value	Cor	*p*-value
CD160	0.3325	**8.74372E-15**	0.06097	0.413573727
CD200R1*	0.24954	**9.14416E-09**	0.17449	**0.018480348**
CD27*	0.17448	**6.7642E-05**	0.23583	**0.001350952**
CD274*	0.40513	**8.40209E-22**	0.34536	**1.79937E-06**
CD276*	0.56566	**5.63722E-45**	0.26113	**0.000370059**
CD40*	0.35322	**1.31282E-16**	0.26368	**0.000322401**
CD40LG	0.28925	**2.10951E-11**	0.14314	0.053899162
CD44*	0.50764	**3.77416E-35**	0.39243	**4.27534E-08**
CD48*	0.35779	**4.99375E-17**	0.31606	**1.38392E-05**
CD80	0.36708	**6.63494E-18**	0.07822	0.293892322
CD86*	0.507	**4.74547E-35**	0.43118	**1.22864E-09**
CTLA4	0.28169	**7.23782E-11**	0.05094	0.494632873
HAVCR2*	0.51212	**7.65559E-36**	0.43182	**1.15445E-09**
ICOS*	0.29238	**1.25147E-11**	0.15868	**0.032396845**
LAIR1*	0.51816	**8.60015E-37**	0.35588	**8.20385E-07**
LGALS9*	0.41799	**3.07881E-23**	0.32819	**6.09713E-06**
NRP1*	0.2512	**7.24034E-09**	0.15429	**0.037555307**
PDCD1	0.32462	**3.9727E-14**	0.12887	0.082960584
PDCD1LG2*	0.52995	**1.05509E-38**	0.53739	**5.2192E-15**
TMIGD2*	0.10197	**0.020515649**	0.18112	**0.014407222**
TNFRSF14*	0.25042	**8.08104E-09**	0.31909	**1.13137E-05**
TNFRSF9*	0.21593	**7.36103E-07**	0.15785	**0.033325864**
TNFSF15*	0.20116	**4.11174E-06**	0.17428	**0.018624787**
TNFSF4*	0.36949	**3.88987E-18**	0.35554	**8.41948E-07**
VTCN1	0.14069	**0.001354566**	0.02874	0.700143724

Values in bold indicate *p* < 0.05, * was considered significant in both TCGA and CGGA.

### 3.4 Correlation between FDX1 and other cuproptosis-related genes

**FIGURE 11:**
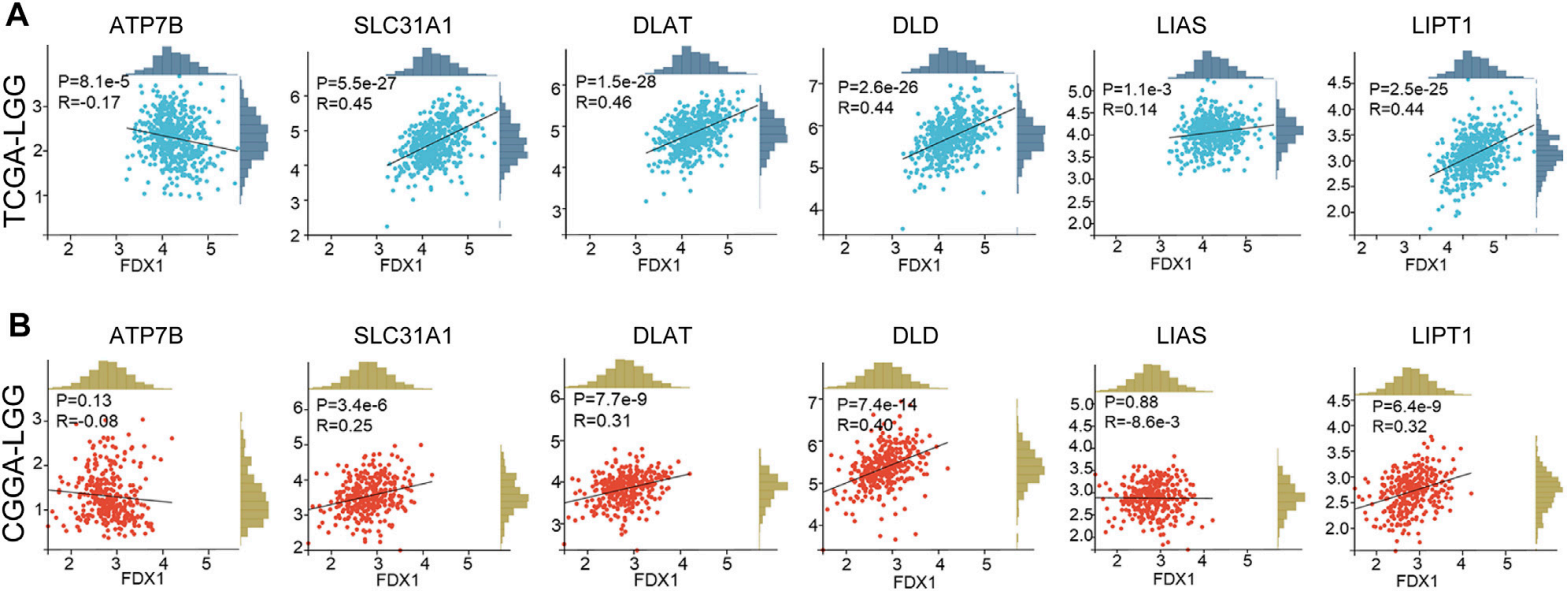
Correlation between FDX1 expression and cuproptosis-related genes. **(A)** The relationship between FDX1 expression and cuproptosis-related genes in TCGA-LGG dataset. **(B)** The relationship between FDX1 expression and cuproptosis-related genes in CGGA-LGG dataset. [Cuproptosis-related genes: ATP7B, SLC31A1, dihydrolipoamide S-acetyltransferase (DLAT), dihydrolipoamide dehydrogenase (DLD), lipoyl synthase (LIAS), and lipolytransferase 1(LIPT1). Correlation analysis: Pearson coefficient, *p* < 0.05 was considered significant.]

Correlation analysis was employed to clarify the relationship between FDX1 and cuproptosis-related genes. Our result suggested FDX1 expression was negatively related to cuproptosis inhibitory gene ATP7B, and positively correlated to cuproptosis-promoting gene SLC31A1, cuproptosis essential genes dihydrolipoamide S-acetyltransferase (DLAT), dihydrolipoamide dehydrogenase (DLD), lipoylsynthase (LIAS), lipolytransferase 1 (LIPT1) in TCGA-LGG dataset (Figure 11A). In the CGGA-LGG dataset, a similar positive correlation was observed between FDX1 expression and SLC31A1, DLAT, DLD, and LIPT1 (Figure 11B). But there was no difference between FDX1 expression and ATP7B and LIAS.

## 4 Discussion

Cuproptosis a newly valued cell death mode similar to ferroptosis, necroptosis, and pyroptosis. Cuproptosis in tumors is still poorly studied and its key regulatory protein FDX1 is also barely researched in tumors. In our study, we investigated the FDX1 expression pattern in TCGA pan-cancer. Abnormal expression of FDX1 was observed in multiple tumors tissues compared to normal tissues in TCGA and GTEx database. FDX1 was upregulated in BLCA, BRCA, CESC, COAD, ESCA, GBM, KICH, LAML, LGG, LIHC, LUAD, OV, PAAD, PRAD, SKCM, STAD, TGCT, THCA, and UCS, and was downregulated in CHOL, KIRC, KIRP, LUSC, and READ (Figure 1B). FDX1 was differentially expressed in different stages in THCA, LIHC, KIRC, PAAD, READ, KIRP, ESCA, and KICH (Figure 2), suggesting a correlation between FDX1 expression and tumor progression. Importantly, survival analysis showed that FDX1 expression predicted poor prognosis in GBMLGG, LGG, and good prognosis in KIPAN, KIRC (Figure 3A). However, the role of FDX1 in predicting prognosis in GBM, KICH, and KIRP was not very significant, indicating FDX1 mainly influenced prognosis in LGG and KIRC. The results demonstrated that FDX1 was correlated with clinical characteristics in multiple tumors and could be a prognostic predictor in LGG and KICH.

Correlations were observed between FDX1 expression and B cell, CD4^+^T cell, CD8^+^T cell, neutrophil, macrophage, and dendritic cell (DC) infiltration in multiple cancers (Figure 4A), suggesting a potential role in the tumor immune microenvironment. Analysis of signatures of immunotherapy prediction including immune checkpoints, tumor stemness, HRD, and TMB also indicated FDX1 could be a potential predictor of immunotherapy in multiple tumor types, especially in LGG. Specifically, most immune checkpoints including CD276 (B7-H3), CD274 (PD-L1), PDCD1LG2 (PD-L2), CTLA4, HAVCR2, and PDCD1 (PD-1) were strongly correlated to FDX1 in LGG (Figure 5A), suggesting high expression of FDX1 was associated with the suppressive immune microenvironment. We also found FDX1 had a prominent positive correlation with tumor stemness, HRD, and TMB in LGG while had an opposite correlation in KIRC (Figures 5B, C; Figure 6A). Tumor stemness is reported to be negatively associated with anticancer immunity (Miranda et al., 2019). And combined PARP inhibition (target HRD) therapy promotes the efficacy of immune checkpoint inhibitors therapy in solid tumors (Peyraud and Italiano, 2020). TMB is an emerging independent predictor of treatment response to immune checkpoint inhibitors for immunotherapy in pan-cancer and high TMB is associated with longer survival after treatment with immune checkpoint inhibitors (Valero et al., 2021). We therefore speculate that FDX1 might be used as one of the markers of immune status to predict tumor immunotherapy response. This might be the underlying mechanism for differential expression of FDX1 in LGG and KIRC leading to different outcomes.

Involvement of FDX1 in the tumor immune microenvironment depends on tumor types. FDX1 expression was positively correlated to B cell, CD4^+^T cell, CD8^+^T cell, neutrophil, macrophage, and dendritic cell (DC) infiltration in multiple tumors including LGG, GBMLGG, PRAD, and KIRC (Figure 4A). ESTIMATE analysis revealed that FDX1 expression was positively correlated to StromalScore, ImmuneScore, and ESTIMATEScore in LGG and GBMLGG (Figures 4B–D) and was negatively correlated in KIRC, KIPAN (Figures 4B–D). That could be a potential reason for highly expressed FDX1 predicting poor prognosis in LGG but good prognosis in KIRC. GSEA analysis of TCGA-LGG and CGGA-LGG based on FDX1 expression revealed that high expression of FDX1 was correlated to immune-mediated pathways, inflammation, metabolism, and cell death-related pathways (Figure 9). But in TCGA-KIRC, FDX1 expression was negatively correlated to immune-mediated pathways such as Regulation of T cell mediated immunity and T cell receptor signaling pathway (Supplementary Figures S5A–C). It suggested that the role of FDX1 in the tumor immune microenvironment depended on tumor types and thus resulting in different outcomes.

Validation of FDX1 in LGG was performed with CGGA. Similar to TCGA-LGG and TCGA-GBM, FDX1 was highly expressed in WHO IV (GBM) compared with WHO II, III (LGG), suggesting that other factors were intervening for FDX1 to affect the survival of tumor patients (Figure 7A). Isocitrate dehydrogenase (IDH) mutation status and 1p/19q co-deletion status are important factors to estimate the diagnosis, prognosis, and treatment condition of glioma (Eckel-Passow et al., 2015). IDH mutant and 1p/19q co-deletion predict better overall survival and better drug sensitivity in glioma patients. FDX1 was highly expressed in IDH wildtype 1p/19 non-codeletion patients (Figures 7B, C), suggesting a differential FDX1 expression in glioma molecular subtypes and the value of survival and drug sensitivity prediction of FDX1. Survival analysis confirmed the above presumption that highly expressed FDX1 predicted poor prognosis in CGGA-LGG (Figure 8A), which was consistent with the survival analysis result in TCGA-LGG (Figure 3C). Immune infiltration analysis and ESTIMATE analysis in CGGA-LGG (Figure 10) obtained similar results in the analyses for TCGA-LGG (Figure 4), validating the strong correlation between FDX1 and the immune microenvironment in LGG. Otherwise, FDX1 expression demonstrated positive correlations to CD274, CD276, HAVCR2, ICOS, and PDCD1LG2 in both TCGA-LGG and CGGA-LGG datasets (Table 1), suggesting the association between FDX1 and suppressive immune microenvironment.

Cuproptosis death occurs through direct binding of copper to the lipid acylated components of the tricarboxylic acid (TCA) cycle, which leads to lipoylated proteins aggregation and subsequent destabilization of Fe-S cluster proteins, leading to proteotoxic stress and ultimately cell death (Peter et al., 2022). In cuproptosis, FDX1 together with DLAT, DLD, LIPT1, and LIAS are cuproptosis essential proteins, and SLC31A1 is a cuproptosis-promoting protein, while ATP7B works as cuproptosis inhibitory protein. Though FDX1 showed a weak correlation to LIAS, a strongly positive correlation was observed between FDX1 and SLC31A1, DLAT, DLD, LIPT1 in both TCGA-LGG and CGGA-LGG (Figure 11). Additionally, ATP7B was negatively related toFDX1 in TCGA-LGG. The results suggested that FDX1 is closely related to cuproptosis-associated proteins in LGG and works through cuproptosis, which might responsible for FDX1 promoting LGG progression. However, the role of cuproptosis in LGG and related mechanisms, as well as the relevance to the immune microenvironment remained to be further explored.

In our study, we explored the role of FDX1 in TCGA human pan-cancer including clinical and immunological characteristics. Specially, we found FDX1 was a poor prognosis predictor and correlated to the immune microenvironment in LGG with the validation of CGGA. FDX1 had a close correlation to the signatures of immunotherapy prediction including immune checkpoints, tumor stemness, HRD, and TMB, which demonstrated its potential role as an immunotherapy predictor. Collectively, our study applied pan-cancer bioinformatics analysis and found that FDX1 might be considered a potential poor prognosis biomarker and immunotherapy predictor in LGG. But all the results above still need further experimental verification.

## Data Availability

The original contributions presented in the study are included in the article/Supplementary Material, further inquiries can be directed to the corresponding authors.
