# Supplementary material for: Pan-cancer integrated bioinformatics analysis reveals cuproptosis related gene FDX1 is a potential prognostic and immunotherapeutic biomarker for lower-grade gliomas
Source: Front Mol Biosci. 2023 Feb 7;10:963639. doi: 10.3389/fmolb.2023.963639 (PMC9941349; doi:10.3389/fmolb.2023.963639)
Supplement: Supplementary file 1 [file Table1.DOCX]

Supplementary Material

# Supplementary Figures and Tables

## Supplementary Figures


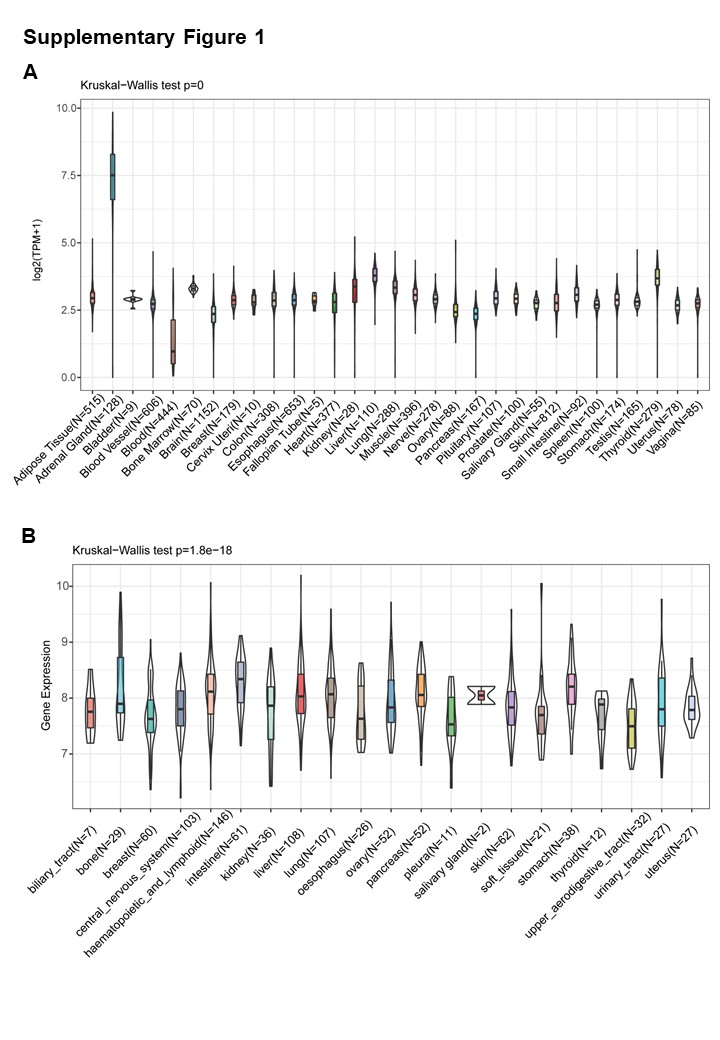


**Supplementary Figure 1.** The mRNA expression of FDX1 in normal tissue based on GTEx database and tumor cell lines based on Cancer Cell Line Encyclopedia (CCLE) database. **(A)** FDX1 expression in normal tissue based on GTEx database. **(B)** FDX1 expression in various tumor cell lines based on CCLE database. (Statistical analysis depended on Kruskal-Wallis test, *P*< 0.05 was considered significant.)


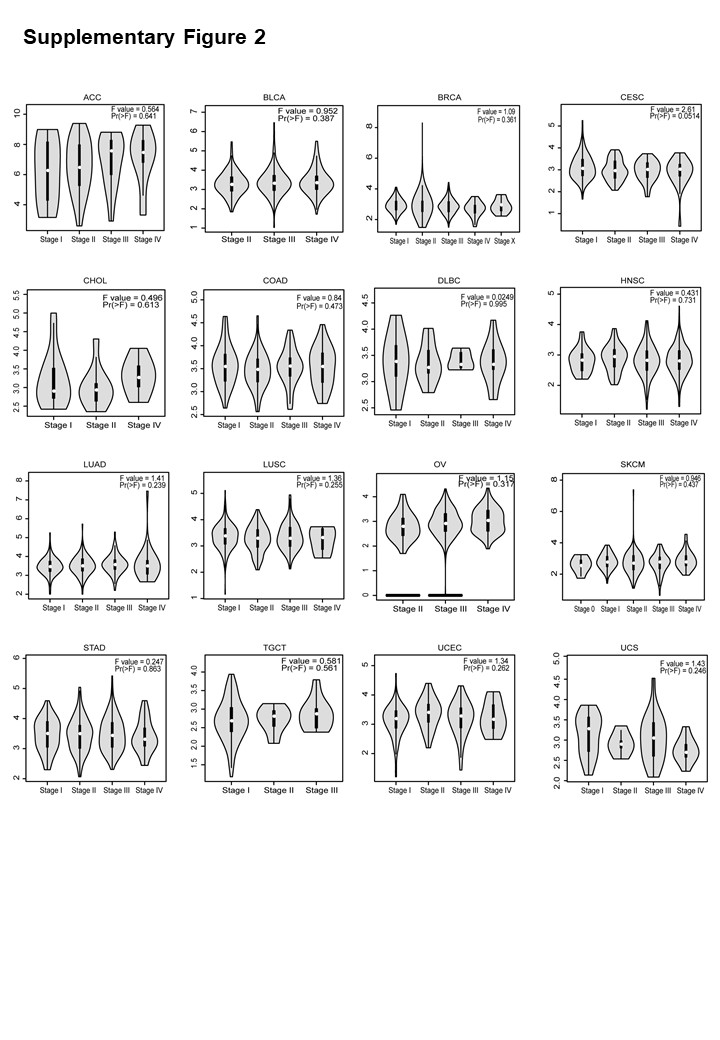


**Supplementary Figure 2.** The relationship between FDX1 expression and tumor stages based on GEPIA2.0. (*P*<0.05 was considered significant)


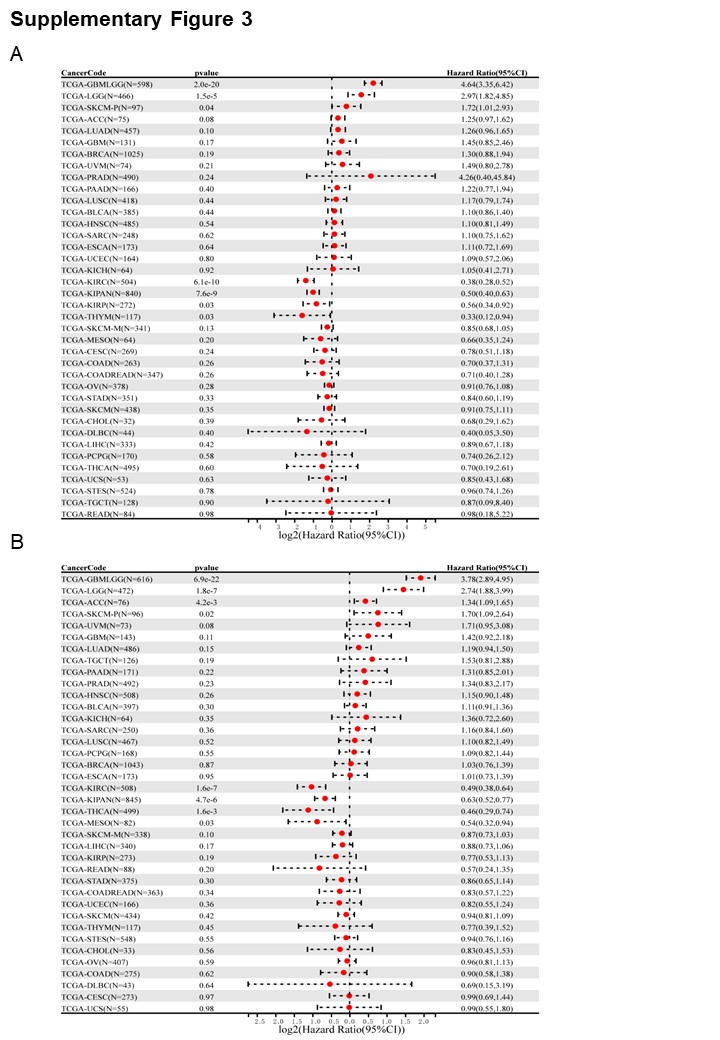


**Supplementary Figure 3.** Expression of FDX1 was associated with disease-specific survival (DSS), progression-free interval (PFI) in human pan-cancer according to the TCGA Pan-Cancer database. **(A)** The forest plot shown the relationship between FDX1 expression and DSS. **(B)** FDX1 expression was related to PFI shown by the forest plot. (HR: hazard ratio, *P*< 0.05 was considered significant.)


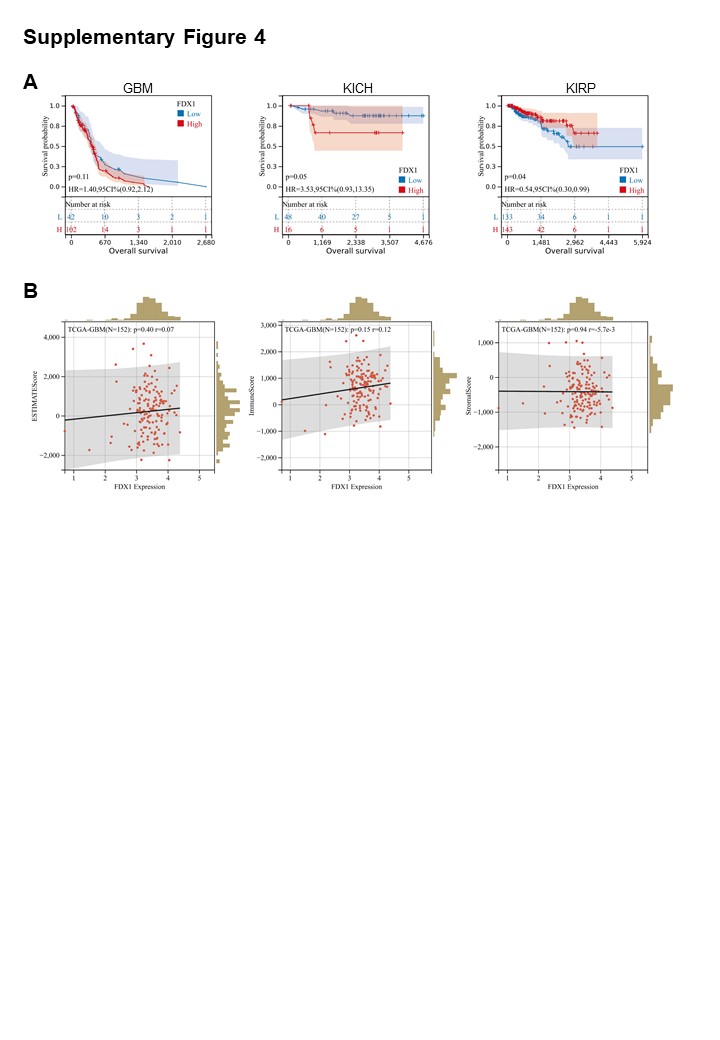


**Supplementary Figure 4.** Correlation between FDX1 expression and OS, ImmuneScore, StromaoScore, and ESTIMATEScore in glioblastoma multiforme (GBM), kidney chromophobe (KICH), and kidney renal papillary cell carcinoma (KIRP). **(A)** Relationship between FDX1 expression and OS in GBM, KICH, and KIRP was shown by Kaplan-Meier plot. **(B)** Relationship between FDX1 expression and ImmuneScore, StromaoScore, and ESTIMATEScore was shown in GBM. (HR: hazard ratio, *P*< 0.05 was considered significant.)


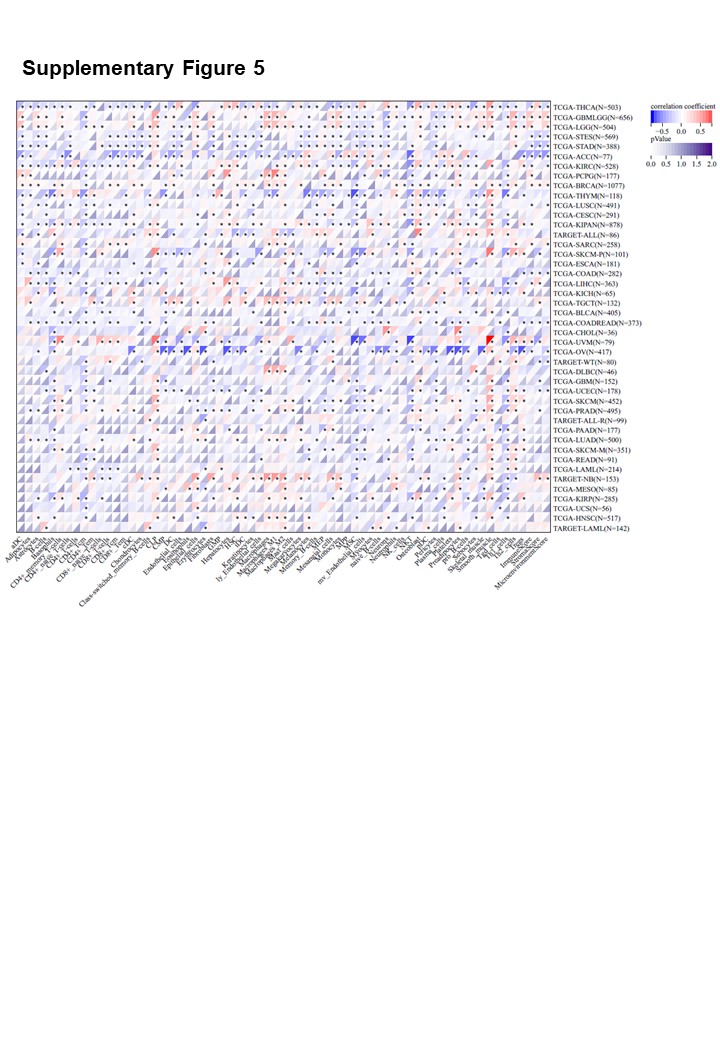
**Supplementary Figure 5** Correlation between FDX1 expression and cell types based on Xcell algorithm in pan-cancer.


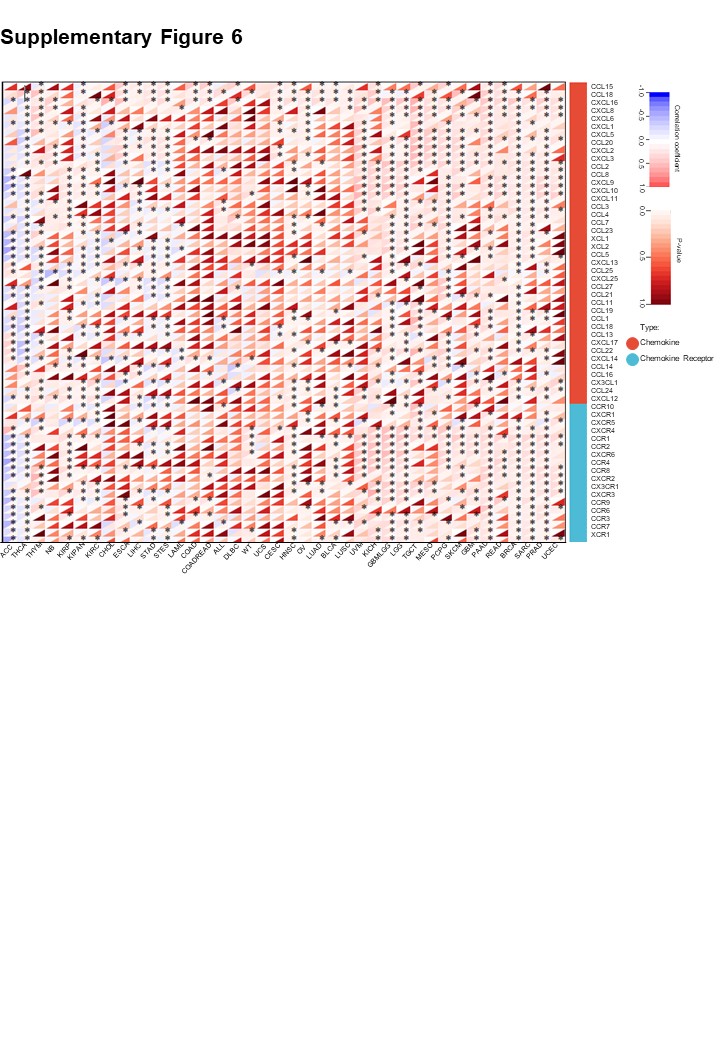
**Supplementary Figure 6** The relationship between FDX1 expression and chemokines and chemoreceptors in pan-cancer.


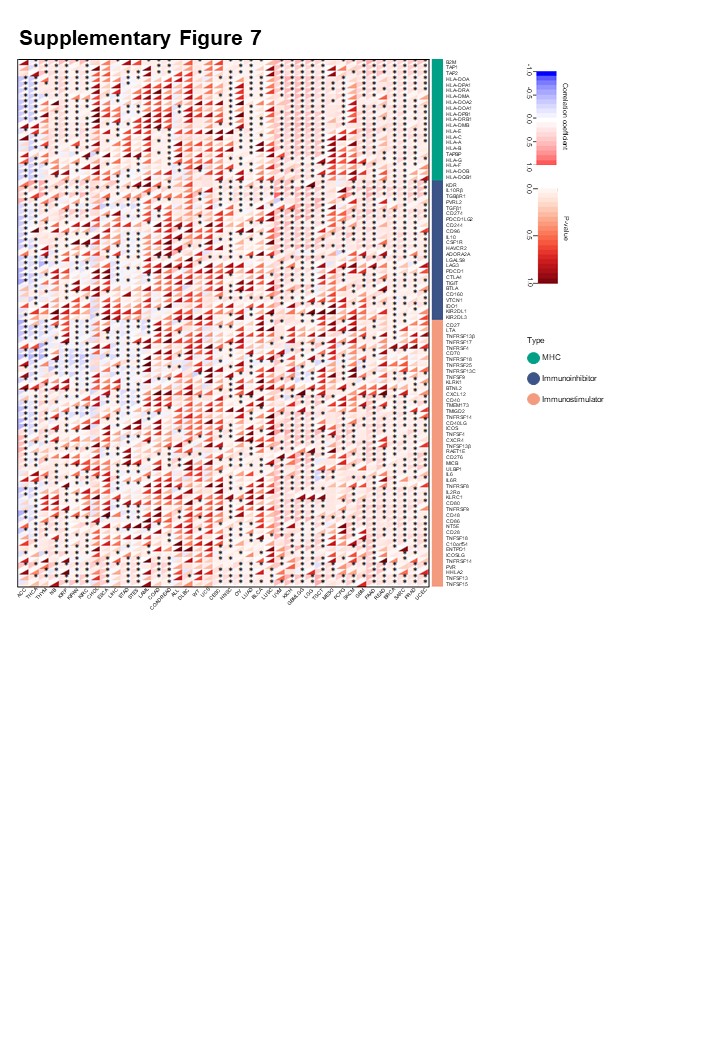
**Supplementary Figure 7** The relationship between FDX1 expression and major histocompatibility complex (MHC), immune-stimulators, and immune-inhibitors in pan-cancer.


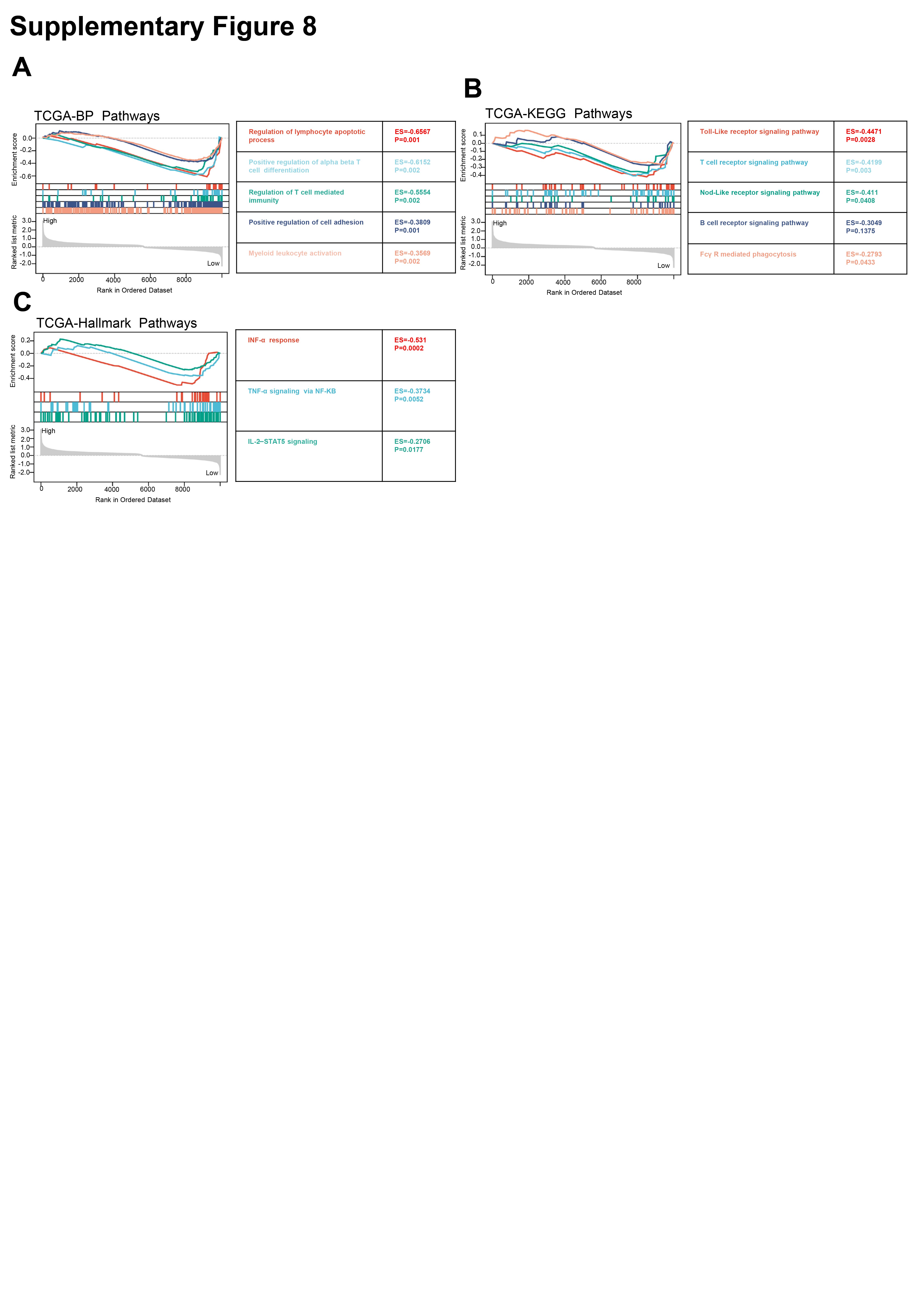
**Supplementary Figure 8.** The biological function of FDX1 gene was shown in biological process (BP) pathways, KEGG pathways, and Hallmark pathways in TCGA-KIRC. **(A)** Relationship between FDX1 expression and BP pathways. **(B)** Relationship between FDX1 expression and KEGG pathways. **(C)** Relationship between FDX1 expression and Hallmark pathways. (All analytical methods have relied on GSEA algorithm, ES: enrichment score, *P*<0.05 was considered significant.)
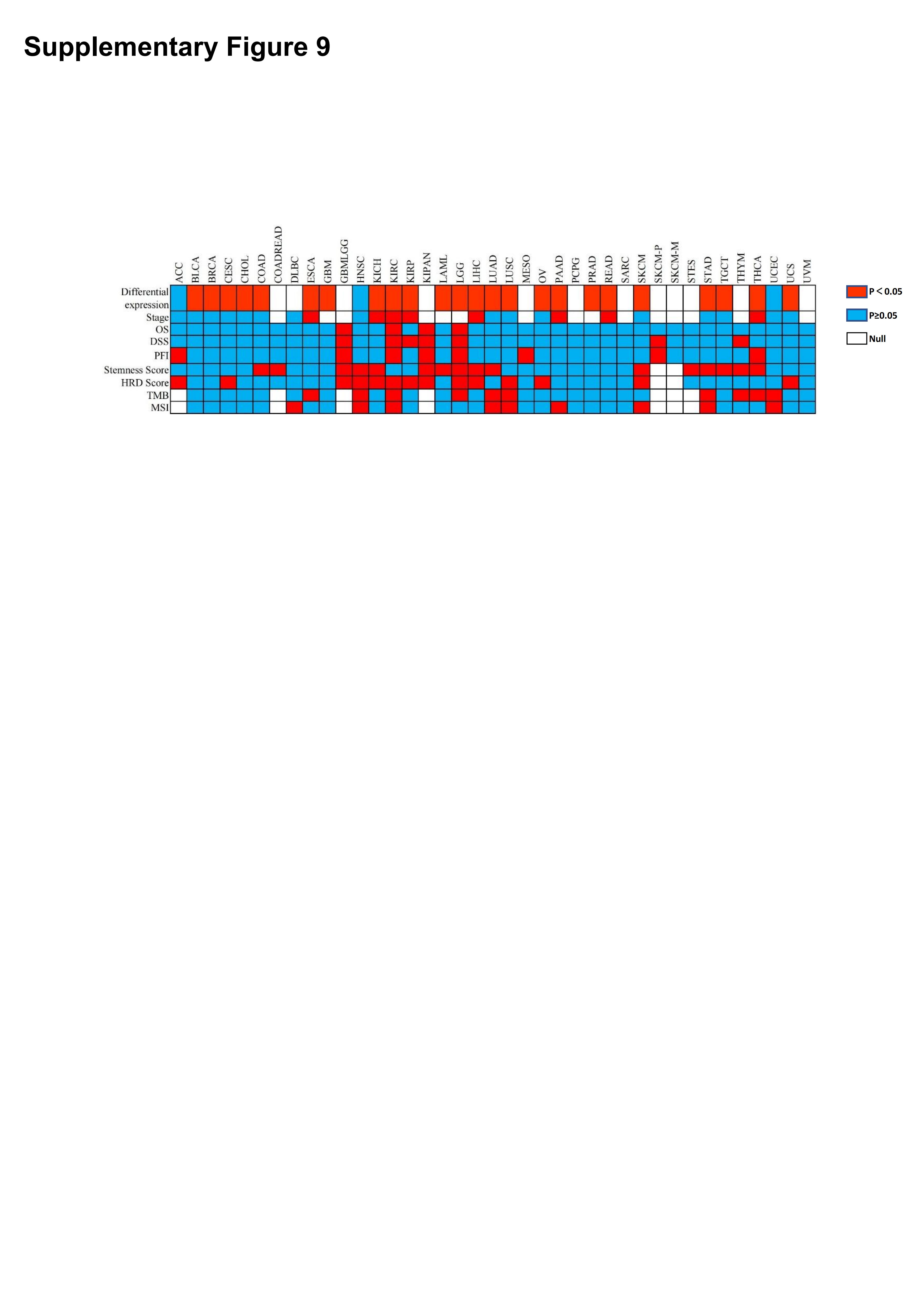
**Supplementary Figure 9.** The overall role of FDX1 in pan-cancer visualized with the main result of pan-cancer analyses in this article. (OS, overall survival; DSS, disease-specific survival; PFI, progression-free interval; HRD, Homologous recombination deficiency; TMB, Tumor mutation burden; MSI, microsatellite instability; Null: data missed.)
